# Clinical features of patients with non-Hodgkin’s lymphoma metastasizing to the pituitary glands

**DOI:** 10.3892/ol.2013.1266

**Published:** 2013-03-19

**Authors:** JUNJIE YANG, NA ZHAO, GUANGSEN ZHANG, WENLI ZHENG

**Affiliations:** Division of Hematology, The Second Xiangya Hospital, Central South University, Changsha, Hunan 410011, P.R China

**Keywords:** non-Hodgkin’s lymphoma, diabetes insipidus, anterior hypopituitarism, pituitary

## Abstract

It is rare for systemic non-Hodgkin’s lymphoma (NHL) to metastasize to the hypothalamus and pituitary glands. The present study describes two patients with NHL and diabetes insipidus (DI) and 17 patients from the literature in order to analyze the clinical features of patients with NHL metastasizing to the pituitary glands. Diffuse large B cell lymphoma (DLBCL) was observed to be the most common type of NHL involving the hypothalamus-pituitary axis. A total of 11 patients (57.9%) had been diagnosed with DI (post-pituitary involvement), five (26.3%) with anterior hypopituitarism and three (15.8%) with posterior and anterior hypopituitarism. Only two cases exhibited simultaneous endocrine and lymphoma manifestations; the majority of cases (68.4%) exhibited lymphoma manifestations first. To make an etiological diagnosis of NHL with metastases to the pituitary glands, it is necessary to find that NHL exists in other regions of patient’s body. Biopsy of the sellar may have significant meaning, but this examination may difficult to perform. Chemotherapy for NHL relieves pituitary impairment symptoms and improves the overall examination results. Additionally, magnetic resonance imaging (MRI) of the pituitary gland has a certain differential diagnostic value as the T1- and T2-weighted imaging (WI) signals from patients with systemic NHL with pituitary involvement are low.

## Introduction

It is rare for systemic lymphoma to involve the hypothalamus-pituitary axis. Out of 380 cases with pituitary tumor metastasis, there were only two cases (0.5%) with malignant lymphomas (including Hodgkin’s lymphoma). The frequency of non-Hodgkin’s lymphoma (NHL) involving the hypothalamus-pituitary axis is <0.5% among malignant pituitary metastases (1). Therefore, it is essential, but difficult, to differentiate pituitary lymphoma metastasis from other tumors to aid the selection of the appropriate therapeutic plan. Aided by a review of the literature, the present study aimed to report our experiences of pituitary metastasis from NHL, summarize the common symptoms of NHL with pituitary metastasis and emphasize the importance of the use of MRI.

## Materials and methods

### Cases

The medical files of two patients diagnosed with diabetes insipidus (DI) caused by pituitary metastasis of NHL, who were admitted in the last five years to the Second Xiangya Hospital (Central South University, Changsha, China), were retrospectively studied (Table I).

### Bone marrow (BM) examination

For patient 1, the BM smear exhibited decreased hyperplasia and one type of immature cells that accounted for 23% of the cells, but whose nature was difficult to determine. The nuclear chromatin of these cells was normal and the nucleoli were observable. The form of the cells was similar to that of a lymphocyte. A BM biopsy showed that one type of primitive cell with an unclear classification was present at increased levels, with a ++ (positive) argyrophil stain. With regard to patients 2, the BM smear showed highly active BM hyperplasia, the lymphocytes accounted for 61.5% of the cells, the lymphoblasts and prolymphocytes accounted for 45% and the cell bodies were large with observable vacuoles. The smear was negative for peroxidase staining, while CD10, CD19, CD20, CD34 and HLA-DR were positive in the leukemic cells, according to flow cytometric detection. By combining these results with the kidney puncture pathology and by considering WHO standards, the diagnosis of patient 2 was that of acute lymphocytic leukemia (ALL; L3, Burkkit’s lymphoma, stage IV).

### Pathological examination

The color doppler flow imaging (CDFI) ultrasound of patient 1 showed that there were multiple hypoechoic nodules in the cervical region and groin, which were identified as lymph nodes, thus biopsies of the left inguinal and right cervical lymph nodes were performed. The pathology of the left inguinal lymph node revealed that the lymph node structure was not clear using microscopy, and that sporadic abnormal cells were present. According to the immunohistochemistry the left inguinal lymph node samples contained CD20^+^ large cells, while CD45RB, CD3, CD45RO, ALK, CD15, CD30 and CD5 were all negative with a positive bcl-2 focal shape (+). Consequently, lymphoma could not be completely ruled out. The pathological results from the right cervical lymph node showed that the lymph node structure was not visible under microscopy due to large amounts of karyopyknosis and necrosis, with only a few slightly degenerated cells left over from the edge, which were similar to lymphocytes with plasma cell-like differentiation. The immunohistochemistry of the right cervical lymph node showed that the samples were only marginally positive for CD138 at the edge. By combining these results with those of the inguinal lymph node section and clinical situation, the patient was diagnosed with lymphatic plasma cell lymphoma.

Patient 2 underwent a kidney puncture biopsy as well as computed tomography (CT) of the chest and abdomen. The pathology report revealed that numerous diffuse infiltrations of the renal interstitium were present with irregular forms and large nuclei. The nuclear staining of the infiltrations was uneven, indicating focal aggregation from multiple sites. Degeneration was also observed among the epithelial cells of the renal tubule and the protein tube was observed in the small lumen. Many cells, with irregular form and large nucleus, were found to infiltrate into the small lumen and tubular wall and nuclear staining of these infiltrating cells were far from uniform. There were destruction and breaking off of the residual basement membrane of the epithelial cells in parts of the renal tubule. Segmental mild proliferation could be found in the mesangial matrix of renal tubule. No obvious fuchsinophilic bodies deposition was found in the mesangium region and the basement membrane was not thick with capillary lumens opening well. In the immunofluorescence results, one glomerulus was observed, IgA^+^ staining was localized in the mesangial area and staining for IgG, IgM, Fib, C3, C4 and C1q was all negative. The pathological diagnosis was of tubulointerstitial lesions that may have been caused by a tumor. The pathology department observed that more lymphocyte infiltration was detected in the renal tissues (mainly the medulla) and that certain lymphocytes infiltrated the renal tubule, causing focal destruction, although the majority of the renal tubular structures were present. In the immunohistochemical evaluation, CD3^+^ and CD20^−^ staining were observed and the majority of the renal tubular structures were present. Consequently, NHL was proposed as the diagnosis of patient 2.

### Water deprivation and vasopressin response test

Urine measurements were obtained from each patient, including the 24-h urine volume (UV), specific gravity (SG), urine osmotic pressure (Uosm) and urine electrolyte levels. Water deprivation and vasopressin (DDAVP) response tests were also performed.

### Imaging

The two patients underwent plain and enhanced MRI at diagnosis and were assessed again following treatment. Other imaging techniques, including CT and single photon emission CT (SPECT) bone scanning were also performed if required.

## Results

### Diagnoses

Based on the clinical characteristics, blood examinations and bone marrow and histological studies, patient 1 was diagnosed with lymphatic plasma cell lymphoma and patient 2 was diagnosed with Burkitt’s ALL (L3, stage IV). The urine measurements, including the water deprivation and DDAVP response tests, revealed that the two patients had developed DI (Table II).

### Imaging

**Figure 1:**
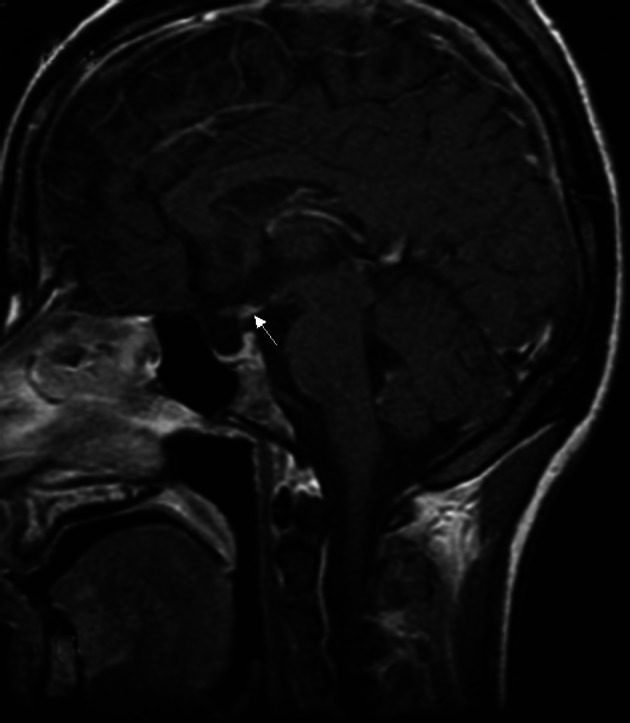
Enhanced magnetic resonance image (MRI) of the sagittal section of patient 1 showing the nodular lesions below the hypothalamus region. The posterior pituitary continued to lack the characteristic high signal.

**Figure 2:**
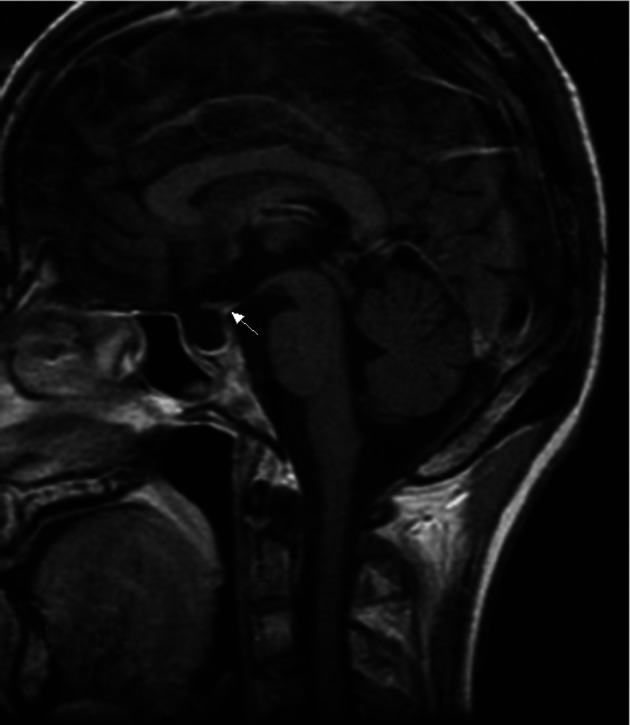
Enhanced magnetic resonance image (MRI) of the sagittal section of patient 1 showing the narrowing of the nodular lesions below the hypothalamus region following treatment.

**Figure 3:**
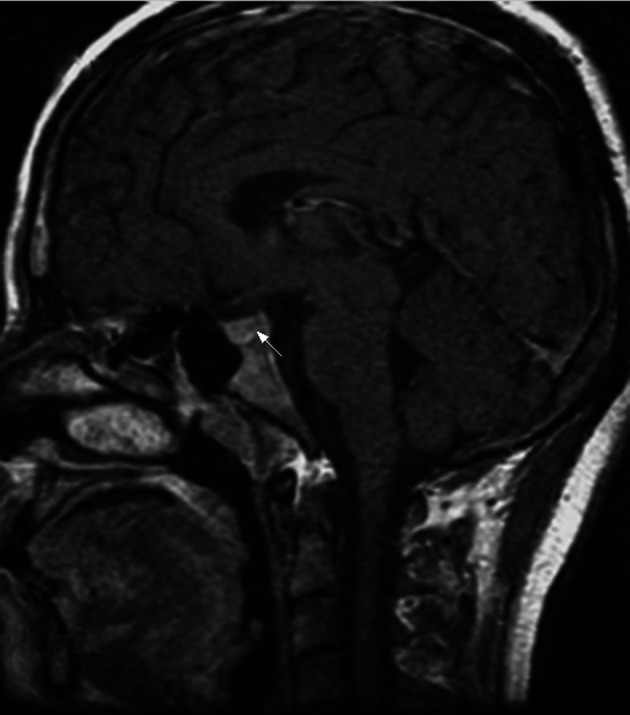
Non-enhanced magnetic resonance image (MRI) of the sagittal section of patient 2 showing the disappearance of the characteristic high signals of the posterior pituitary and increased size of the pituitary stalk.

**Figure 4:**
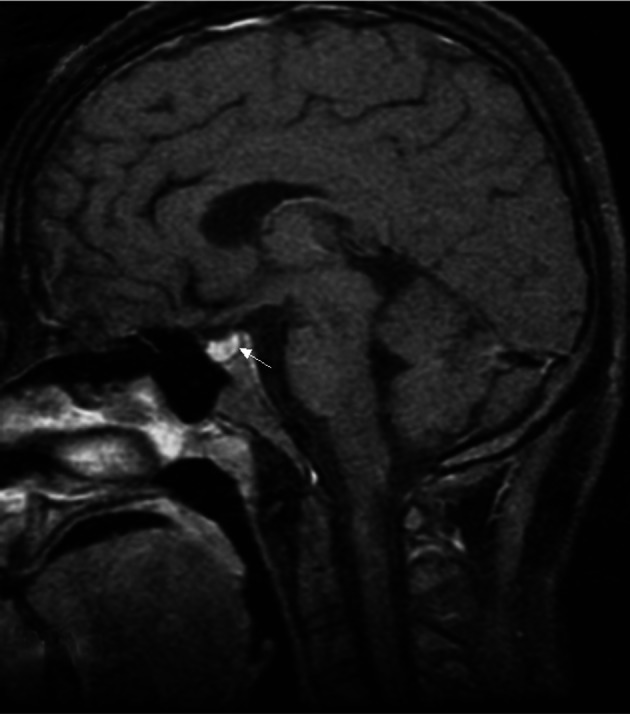
Enhanced magnetic resonance image (MRI) of the sagittal section of patient 2 showing the narrowing of the pituitary stalk following treatment. The posterior pituitary continued to lack the characteristic high signal.

Plain and enhanced MRI examinations of the pituitary glands were performed at diagnosis and following treatment for each patient (patient 1, Figs. 1 and 2; patient 2, Figs. 3 and 4). With regard to patient 1, intensified plain MRI scanning of the pituitary revealed a small nodular lesion under the hypothalamus (tuber cinereum) and the disappearance of the normal high signal from the posterior pituitary, suggesting that there was infiltration and obstruction in the hypothalamic portal system caused by lymphoma or other pathological changes. Additionally, in the lumbar vertebral MRI of patient 1, numerous vertebral body changes were identified as malignant lesions that were possibly due to tumor metastasis. SPECT bone scanning also showed bone destruction in the skull, fifth rear-right rib, third lumbar vertebra, left sacroiliac joint and right acetabulum.

The MRI of the patient 2 revealed that the hypophyseal fossa was small and the high signals from the posterior pituitary had disappeared. Notably, enhanced CT of the chest and abdomen showed bad bilateral perfusion in the kidneys that may have been associated with diffuse and infiltrative tumors.

### Treatment

The two patients were diagnosed with NHL and DI caused by NHL pituitary metastases and were treated with DDAVP and chemotherapy.

For patient 1, 0.1 mg DDAVP was administered twice a day and then increased to three times per day after five days. The patient also underwent the cyclophosphamide, epirubicin, vincristine and prednisone (CHOP) chemotherapy regimen. After 20 days, the patient’s UV decreased, so the DDAVP dose was reduced to 0.1 mg twice a day. Subsequent to one month of chemotherapy, all the patient’s swollen superficial lymph nodes were not palpable and the blood index improved noticeably. A second cycle of CHOP was subsequently administered. The patient also underwent cranial radiation therapy using a linear accelerator five times a week for one month. Later, the patient received the third, fourth, fifth and sixth CHOP regimes as monthly chemotherapy. Reexamination with plain and enhanced MRI scanning of the pituitary gland showed that the nodular enhanced focus in the hypothalamus (tuber cinereum) was smaller than at diagnosis, but there was no clear change in the other foci (Fig. 2). No enlargement was observed in the retroperitoneal lymph node. Routine blood tests, kidney and liver function tests, blood sedimentation rate and lactate dehydrogenase levels were all normal. The patient received 0.1–0.15 mg DDAVP per day to maintain a normal UV.

Patient 2 underwent the cyclophosphamide, epirubicin, vincristine, etoposide and prednisone (EPOCH) regimen. The patient started receiving DDAVP (0.2 mg, twice per day) two days after the beginning of chemotherapy. Two weeks afetr chemotherapy, the polydipsia disappeared and the patient’s UV dropped from 7,000–8,000 ml to 2,400 ml per day. The SG was 1.018 and the Uosm was 680 mOsm/l. The patient’s blood LDH was 342.1 U/l. DDAVP was gradually reduced to avoid withdrawal. A BM reexamination revealed complete remission. A brain MRI showed that the pituitary stalk had returned to normal but that the normal high signal of the posterior pituitary remained unobservable (Fig. 4). Two months later, following the second EPOCH course, the disease relapsed. The patient’s UV increased to 4,000 ml per day and the SG and Uosm were 1.007 and 300 mOsm/l, respectively. Lymphoblasts and prolymphocytes in the BM accounted for 24% of all cells. Cyclophosphamide, pirarubicin, vinorelbine, etoposide and prednisone were administered as chemotherapy in the third and fourth courses, in addition to 0.1 mg oral DDAVP twice a day. The UV, SG and Uosm were controlled within the normal range, but the lymphoblasts and prolymphocytes of the BM continued to account for 14% of the cells. In the fifth and sixth chemotherapy courses, itoxantrone, cytarabine and dexamethasone were administered, as well as 100 mg oral thalidomide per day for 21 days each month. The patient remained in excellent physical condition despite continuously presenting with 12.5% prolymphocytes in the BM.

The present study also reviewed 17 cases from the literature. The clinical features of these cases, as well as the two cases described in the present study, are reviewed in Table III.

## Discussion

The BM analysis, histological results of other regions and the observed efficiency of chemotherapy aid in the diagnosis of NHL. With regard to the two patients studied retrospectively in the present study, the NHL diagnoses were identified by the histology and BM examinations. However, NHL is a systemic disease with various clinical manifestations, including endocrine symptoms. Occasionally, the endocrine symptoms precede the hematological diagnosis. Therefore, malignant lymphomas should be systematically considered as a potential etiology of endocrine disease such as DI (3). In the two present cases, the patient’s clinical features and urine measurements, particularly the water deprivation and DDAVP response tests, indicated a diagnosis of DI but not NHL.

Furthermore, the two patients’ clinical conditions were clearly improved following treatment, shown by a remission of polyuria and polydipsia and the normalization of UV, SG and Uosm in the urine reevaluation. This demonstrated that the diagnosis of DI was correct. It is essential that 24-h UV, Usom, plasma electrolytes, formal water deprivation and DDAVP response tests are performed, as well as random plasma osmolality tests to ensure a correct diagnosis (3). However, among the cases from the literature, there was only one patient who had been diagnosed with DI by determining the DDAVP in the plasma (4). The major causes of DI, particularly central DI, are neoplastic or infiltrative lesions of the hypothalamus or pituitary glands, severe head injures and pituitary or hypothalamic surgery (3), therefore, brain radiography is required to identify the causes of DI. MRI and CT have important roles; MRI is the modality of choice for providing multiplanar high-contrast images, whereas CT has a complementary role in delineating bone destruction and the visualization of calcification. MRI is more sensitive than CT in revealing brain lymphoma and is consequently essential (5).

In addition to the two cases reported in the present study, the features of 17 cases from the literature were also reviewed (Table III). The median age of these patients was 52 years (range, 19–77 years). With regard to the histological types, 12 cases had been diagnosed with B-cell NHL, three with angiocentric T cell lymphoma (ACTL), one with follicular lymphoma (FL), one with lymphoplasmacytoid lymphoma (LPL), one with Burkkit’s lymphoma and one was not precisely classified. Patient 1 of the present study is the first reported case of lymphoplasmacytoid lymphoma metastasizing to the pituitary. Among the 19 patients with pituitary function impairment, this was manifested as DI (posterior pituitary involvement) in 11 cases (57.9%), while five cases (26.3%) only had anterior pituitary hypofunction and three (15.8%) had DI and anterior pituitary hypofunction. The diagnoses of the cases were made according to the existence of lymphoma in other parts of the body and the efficiency of chemotherapy. Only one case underwent a biopsy of the sellar region (12). Lymphoma symptoms occurred first in 13 cases and the endocrine symptoms appeared between several days and nine months later. The endocrine symptoms appeared first in four cases and the lymphoma manifestations appeared between six weeks and three years later. Only two cases experienced the endocrine and lymphoma symptoms simultaneously. Among the four patients who experienced endocrine symptoms first, three exhibited posterior pituitary involvement and one was diagnosed with lymphoma following a three-year anterior pituitary function impairment. In total, three cases with ACTL exhibited posterior pituitary involvement. Komninos *et al* reviewed 190 cases with systemic malignant pituitary metastases and of these, 86 cases (45.2%) developed DI due to posterior pituitary impairment, while 45 cases (23.6%) exhibited anterior pituitary function impairments (1). Among the 19 cases from the present literature review, the two cases reported in the present study and 12 other cases from the literature exhibited posterior pituitary impairments, accounting for 73.7% of the cases (14/19), while eight had anterior pituitary function impairment, accounting for 42.1%. The frequency of certain symptoms in this review did not precisely correlate with those of the published literature (1), with the exception of statistical differences due to the limited number of cases, although they may be relevant in identifying the different sources of the initial tumor (NHL vs. all malignant tumors). DI due to posterior pituitary impairment or compression of the pituitary stalk is the most common manifestation of systemic NHL involved with the pituitary glands. The neurohypophysis receives its blood supply directly from the pituitary artery, while the anterior pituitary receives blood from the portal system and a branch originating from the posterior pituitary. This difference in blood supply is the reason why the posterior pituitary is more easily impaired than the anterior pituitary.

The changes that are observable using MRI in patients with systemic malignant pituitary metastasis have similar presentations, including intrasellar and parasellar destructive and nonhomogeneous enhanced impairments, often affecting adjacent structures. Normal pituitary cells contain phospholipids or secretory granules, so the T1-weighted imaging (WI) signal of MRI is enhanced. In DI, during the hypofunctioning of pituitary synthesis, transport and storage, this signal is weakened or disappears and there is homogeneous enhancement of the pituitary and pituitary stalk signals following the administration of contrast agent. The MRI of patients with lymphoma shows low T1WI and T2WI signals (5,19–21), while for pituitary metastases from other tumors, T1WI signals are usually low and T2WI signals are high (5). Among the 14 cases with posterior pituitary involvement, 11 patients underwent MRI and two underwent only CT. The disappearance of the normal higher signals in the posterior pituitary occurred in four cases (4/11; 36.4%). Enlargement of the pituitary gland and pituitary stalk thickening occurred in four cases (4/13; 30.8%). There was significant diversity in anterior pituitary involvement, including the occurrence of suprasellar masses, destruction at the base of the sella, optic chiasm infiltration, cavernous sinus masses, clival damage and leptomeningeal and pituitary masses.

A number of the NHL cases first manifested as endocrine symptoms. The NHL pituitary metastasis rate was <0.5%. Consequently, it is difficult to identity the causes of pituitary impairment, particularly NHL metastasis to the pituitary. Unless a lesion biopsy of the sellar region is performed or there is evidence of a pituitary gland tumor, it is difficult to distinguish NHL pituitary impairment from the pituitary metastasis of other tumors. Even an image-guided biopsy is unable to reliably avoid the surrounding critical neurovascular structures since the sella is a region with a small volume in close proximity to numerous complex structures (5). In the literature review, only one patient underwent this type of biopsy. Therefore, it is important and necessary to perform pituitary MRI to reveal pituitary metastases from NHL. Moreover, chemotherapy for certain tumors that relieves pituitary impairment symptoms and improves the imaging results is useful for an etiological diagnosis.

In summary, in our two cases, diabetes insipidus is their main and early clinical manifestation. Thus, for patients exhibiting endocrine symptoms, NHL should be considered as a potential cause, particularly if hematological symptoms also exist. It is essential to perform pituitary MRI to differentiate NHL pituitary metastases from other tumors. The T1WI and T2WI signals are low in patients with malignant lymphoma involving the pituitary glands, while for pituitary metastases from other tumors, the T1WI signals are usually low and T2WI signals are high. For patients with NHL, the diagnosis of DI depends on water deprivation and DDAVP tests, although plasma DDAVP tests are not necessary.

## Figures and Tables

**Table I:** Patient characteristics at diagnosis.

				Blood measurements				
Patient	Gender	Age (years)	Symptoms	Routine bloods	Kidney function	Electrolyte (mmol/l)	Sugar	Other data
1	Male	20	Body aches, high fever, weight loss, polyuria, polydipsia	Hb, 95 g/l	Normal	Na, 123.8 Others normal	Normal	Albumin, 31.3 g/l LDH, 768.2 U/l ESR, 109 mm/h Ferritin, 1,000 ng/ml
2	Male	26	Polyuria, polydipsia, protienuria	Hb, 62 g/l	Normal	Na, 152.4 K, 2.80 Cl, 127.7 Ca, 1.16 Mg, normal P, normal	Normal	Albumin, normal LDH, 674 U/l

Hb, hemoglobin; Na, sodium; LDH, lactate dehydrogenase; ESR, erythrocyte sedimentation rate; K, potassium; Cl, chloride; Ca, calcium; Mg, magnesium; P, phosphorus.

**Table II:** Urine measurements.

	At diagnosis			After water deprivation			After administration of DDAVP		
Patient	UV (ml)	SG	Usom (mOsm/l)	UV (ml)	SG	Usom (mOsm/l)	UV (ml)	SG	Usom (mOsm/l)
1	10,000	DL	DL	8,000	1.007	268	DL	1.017	470
2	8,000	1.007	300	11,000	1.007	200	2,400	1.017	610

DL, data lost; DDAVP, vasopressin; UV, urine volume; SG, specific gravity; Usom, urine osmotic pressure.

**Table III:** Features of patients with pituitary metastasis of NHL.

Reference	Gender	Age (years)	Type	Involvement region	Imaging	Sequence of lymphoma and endocrine symptoms
2	Male	59	DLBCL	Anterior lobe	MRI; lump in pia mater	Headache 3 weeks prior
6	Female	72	Large B-cell NHL	Anterior lobe	MRI; infiltration of the pituitary and pituitary stalk	Occurred simultaneously
7	Female	70	FL, transformed to DLBCL	Anterior lobe	NR	Follicular manifestation occurred first
8	Male	65	NHL	Anterior lobe	Gallium 67; pituitary tumor	Ophthalmic signs 2 months prior
9	Male	65	B-cell NHL	Anterior lobe	MRI; enlargement of the pituitary gland	Endocrine symptoms 3 months prior
10	Male	77	DLBCL	Anterior, posterior lobe	MRI; the pituitary mass	Occurred simultaneously
11	Male	55	DLBCL	Anterior, posterior lobe	CT and MRI; optic chiasm infiltration	NR
12	Female	50	DLBCL	Anterior, posterior lobe	CT and MRI; suprasellar mass	Lymphoma in the first half of the year
2	Male	53	T cell-rich B cell NHL	Posterior lobe	MRI; the high signal point from the pituitary disappeared	Headaches after 6 weeks of and polydipsia
3	Male	32	Large B-cell NHL	Posterior lobe	Gallium 67; cavernous sinus infiltration	Diabetes insipidus 3 months ago
4	Female	48	DLBCL	Posterior lobe	MRI; no abnormality in the pituitary with encephalitis	Lymphoma 1 month prior
13	Male	19	ACTL	Posterior lobe	CT; pituitary stalk thickening with hypothalamus involved	Lymphoma several days ago
14	Female	56	ACTL	Posterior lobe	MRI; the high signal point from the pituitary disappeared	Lymphoma 3 months prior
15	Male	50	ACTL	Posterior lobe	CT; pituitary stalk thickening, empty sella tarcica	Lymphoma 3 months prior
16	Male	64	Large B-cell NHL	Posterior lobe	MRI; involvement of the sella turcica with pituitary body and pituitary stalk thickening	Lymphoma 2 years prior
17	Female	37	DLBCL	Posterior lobe	MRI; infiltration of the neuro-hypophyseal lymphoma	Lymphoma 3 months ago
18	Female	70	B-cell NHL	Posterior lobe	MRI; a sellar mass involving the pituitary, infundibular stalk, right cavernous sinus and sphenoid sinus	Right palpebral ptosis for 1 week
Patient 1	Male	20	LPL	Posterior lobe	MRI; hypothalamic focus with the disappearance of the high signal from the posterior lobe	Lymphoma 9 months prior
Patient 2	Male	26	Burkkit’s lymphoma	Posterior lobe	MRI; the high signal from the posterior lobe disappeared	Polyuria and polydipsia occurred first

NHL, non-Hodgkin’s lymphoma; FL, follicular lymphoma; ACTL, angiocentric T cell lymphoma; LPL, lymphoplasmacytoid lymphoma; NR, not reported; DLBCL, diffuse large B cell lymphoma; CT, computed tomography; MRI, magnetic resonance imaging.
